# A Highly Sensitive Fiber-Optic Fabry–Perot Interferometer Based on Internal Reflection Mirrors for Refractive Index Measurement

**DOI:** 10.3390/s16060794

**Published:** 2016-05-31

**Authors:** Xuefeng Li, Yujiao Shao, Yuan Yu, Yin Zhang, Shaowen Wei

**Affiliations:** College of Electronic and Information Engineering, Tongji University, Shanghai 201804, China; 5_shao_yu_jiao@tongji.edu.cn (Y.S.); 14yuyuan@tongji.edu.cn (Y.Y.); 1152623@tongji.edu.cn (Y.Z.); 1252781@tongji.edu.cn (S.W.)

**Keywords:** FFPI sensor, micro-analysis technology, refractive index, open micro-cavity, gold thin films

## Abstract

In this study, a new type of highly sensitive fiber-optic Fabry–Perot interferometer (FFPI) is proposed with a high sensitivity on a wide refractive index (RI) measurement range based on internal reflection mirrors of micro-cavity. The sensor head consists of a single-mode fiber (SMF) with an open micro-cavity. Since light reflections of gold thin films are not affected by the RI of different measuring mediums, the sensor is designed to improve the fringe visibility of optical interference through sputtering the gold films of various thicknesses on the inner surfaces of the micro-cavity, as a semi-transparent mirror (STM) and a total-reflection mirror (TRM). Experiments have been carried out to verify the feasibility of the sensor’s design. It is shown that the fabricated sensor has strong interference visibility exceeding 15 dB over a wide measurement range of RI, and the sensor sensitivity is higher than 1160 nm/RIU, and RI resolution is better than 1.0 × 10^−6^ RIU.

## 1. Introduction

The refractive index (RI) is a key parameter for characterizing of an analyte, and has been widely applied in biomolecule detection for clinical diagnosis, pharmaceutical and drug analysis, pollution control and monitoring [1,2,3]. In recent years, various kinds of fiber-optic sensors have been studied intensely in biomedical measurement and environmental protection, due to their distinct advantages, such as corrosion resistance, immunity to electromagnetic interference, high precision, compactness, *etc*. [4,5]. Fiber-optic sensors, including fiber-optic Fabry–Perot interferometric (FFPI) [6,7,8], fiber Bragg gratings (FBG) [9,10], long period fiber gratings (LPFG) [11,12,13], fiber surface plasmon resonance (SPR) [14,15,16], and other types based on specialty fibers [17,18,19,20], have high sensitivity to RI measurement as their most important advantage. In general, RI sensors, based on the response of resonance wavelength, can obtain high sensitivities and good resolutions for RI measurement. It is noted that the type of RI sensors based on an optical microfiber can provide ultrahigh sensitivity up to 18681.82 nm/RIU and better resolution with 5.35 × 10^−7^ RIU [17]. However, these types of sensors have some downsides, such as temperature cross-sensitivity and complex structures. Especially, these kinds of sensors can only be applied to measuring mediums whose RI has to be less than that of the optical fiber, which restricts their practical applications [21]. Meanwhile, SPR sensors have similar problems with temperature cross-sensitivity. In addition, SPR sensor systems are expensive, due to their incompatibility with the near-infrared (NIR) optical communication technology [22].

FFPI sensors have the most attractive characteristics, such as high sensitivity, linear response, small size, and small temperature cross-sensitivity. Best of all, the phase of the interference signal is linearly proportional to the optical length, defined as the product of the micro-cavity length and the RI of the filling medium. Therefore, FFPI sensors have been widely used for measurement of a variety of parameters such as RI, temperature, strain, *etc*. [23,24,25,26]. Our team proposed an FFPI sensor based on the difference in the thermal expansion coefficient between fused silica and metallic materials, which has a high temperature sensitivity with 70 pm/°C [25,26]. Again, notice that the optical length of the micro-cavity is easier to change by the RI of the filling medium. The FFPI sensor can be used for high sensitivity RI measurement, and its opened micro-cavity is suitable for microanalysis technology [6,7,8]. However, these types of sensors have ignored one problem that needs to be overcome. When the RI of the measuring medium is close to that of optical fiber, the reflected light from the surface is very weak according to Fresnel law [27]. In this case, the FFPI sensor shows very poor optical performance, and the accuracy and resolution of the RI measurement become deteriorative accordingly [6]. In order to solve this problem, Rao *et al.* reported a laser-machined FFPI sensor for the measurement of an arbitrary RI. However, the accuracy of the intensity-modulation fiber-optic sensor was limited by the performances of the light source, photodetector, and electric circuit, *etc.* [21,22].

Recently, Wieduwilt, *et al.* reported a reflectivity enhanced RI sensor based on a fiber-integrated Fabry–Perot resonator [28]. In this paper, coating the resonator walls with HfO_2_ led to a strongly improved mirror reflectivity by a factor of about 26. Pevec and Donlagic also reported the fabrication of a high reflection mirror in a resonator [29]. TiO_2_ coating layer provides a high reflection when the sensor head is immersed into mediums with various RIs. However, these proposed sensors have a complex structural and production process. Furthermore, coating layers have the same thickness designation, thus the design of transmission and the reflection model is restricted.

In this study, a new type of highly sensitive FFPI sensor is presented. A micro-cavity is directly machined on a single-mode fiber, and then gold films of various thicknesses are sputtered on the inner surfaces of the micro-cavity, as a semi-transparent mirror (STM) and a total-reflection mirror (TRM) [30,31,32]. Therefore, light reflections of the gold thin films will not be affected by the RI of the measuring medium. The main contributions of this paper are listed as follows:

(1) A new type of structure of the FFPI sensor is proposed with high sensitivity on a wide RI measurement range, including RI around that of optical fiber;
(2) A small amount of the analyte is needed for the proposed FFPI sensor as an open micro-cavity, which is used to accommodate the measuring media;
(3) Moderate requirements for detection setup are needed for the proposed FFPI sensor due to a clear fringe visibility of optical interference and a narrower operating wavelength range;
(4) The proposed FFPI sensor has small temperature cross-sensitivity, and the effect from the temperature change can be almost eliminated by a compensated calculation method;
(5) The good chemical stability characteristic of the silica fiber and gold films makes the proposed FFPI sensor suitable to be used in applications prone to chemical reaction.

This paper is organized as follows. In Section 2, design of the sensor and principle analysis are presented. Experimental verifications are given in Section 3, including production and evaluation of gold thin film, fabrication of sensor head, and detailed experimental setup and results. Section 4 follows with a discussion. Finally, Section 5 draws conclusions and outlines future perspectives.

## 2. Design of Sensor and Principle Analysis

**Figure 1:**
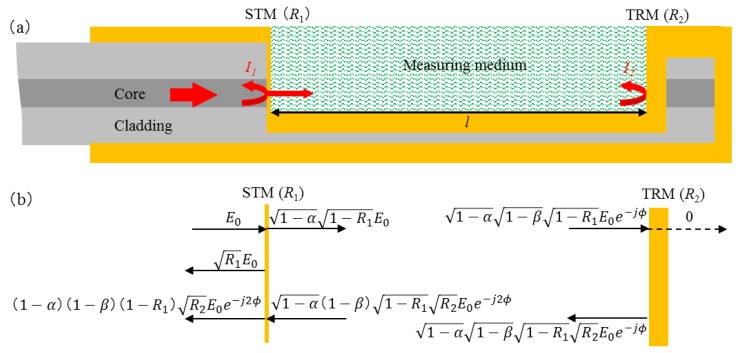
(**a**) The schematic diagram of the FFPI sensor head, showing that the gold films of various thicknesses (yellow part) are sputtered on the inner surfaces of the micro-cavity as a STM and a TRM in the direction of optical axis; (**b**) transmission and reflection model.

The schematic diagram of the proposed sensor is shown in Figure 1a. It is made by a single-mode fiber (SMF). An open micro-cavity is machined, and then gold thin films of various thicknesses are sputtered on the inner surfaces of the micro-cavity. These gold thin films are used as an STM and a TRM in the direction of optical axis. As shown in Figure 1b, the reflection coefficients of two reflective surfaces are *R*_1_ and *R*_2_, respectively.

In addition, the normalized total reflective intensity *I_Rtotal_*(*λ*) can be obtained as follows [6]:

(1) $$I_{Rtotal}(\lambda )={\left|\frac{E_{r}}{E_{0}}\right|}^{2}=R_{1}+{\left(1-\alpha \right)}^{2}\cdot {\left(1-\beta \right)}^{2}\cdot {\left(1-R_{1}\right)}^{2}\cdot R_{2}+2\sqrt{R_{1}\cdot R_{2}}\cdot \left(1-\alpha \right)\cdot \left(1-\beta \right)\cdot \left(1-R_{1}\right)\cdot \cos\left(2\phi \right)$$

where

(2) $$\phi =\frac{2\pi \cdot n\cdot l}{\lambda }$$

Here, *E_0_* and *E_r_* are the input field and the reflective field, respectively; *α* is the loss factor of the transmission at STM; *β* is the loss factor of the transmission through a one-way micro-cavity; *ϕ* is the round-trip propagation phase shifts; *n* is the RI of the measuring medium filling the micro-cavity and its unit is RIU; *l* is the length of the micro-cavity; and *λ* is the optical wavelength. Equations (1) and (2) describe the interference spectrum of the total light reflected from the sensor head.

In addition, please note that the proposed FFPI sensor is designed based on the simplified model in this work. Thus, small errors exist because plane waves are used to analyze two-beam interference. However, the simplified model is widely adopted to design and analyze the FFPI sensor due to its effectiveness and simplicity in practical applications with careful parameters selection [6,7,8].

The interference pattern of a normalized total reflective intensity is investigated firstly. The design parameters are set as *R*_1_ = 0.1, *R*_2_ = 1.0, *α* = 0.5, *β* = 0.5, and *l* =100 µm. Here, *R*_1_, *R*_2_, and *α* are designed to obtain a clearer fringe visibility of optical interference. The loss factor *β* is calculated from loss analysis of single-mode fiber splices by Equation (3) [33,34]:

(3) $$\beta =1-T_{s}=1-{\left[1+{\left(\frac{\lambda \cdot l}{\pi \cdot n_{mc}\cdot \omega^{2}}\right)}^{2}\right]}^{-1}$$

Here, *T_s_* is a transmission coefficient of spliced single-mode fibers, *n_mc_* is the RI of the medium filling the micro-cavity, and ω is a mode field radii of the SMF.

**Figure 2:**
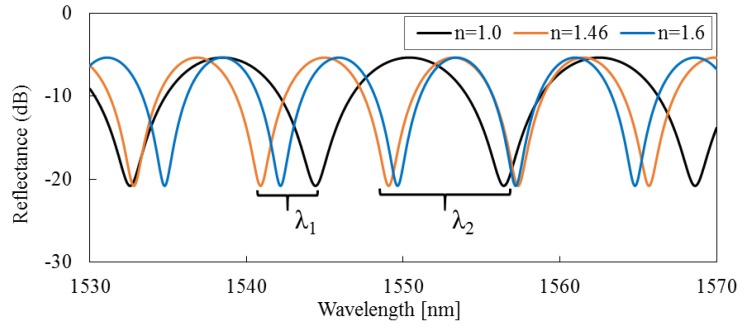
Reflection spectra calculated for RI of *n* = 1.0 (less than RI of silica), *n* = 1.46 (approximately equal to RI of silica), and *n* = 1.6 (greater than RI of silica), respectively.

Figure 2 shows the interference spectra of the calculated results by Equations (1) and (2). Because the RI of silica optical fiber is near 1.46, the RIs of input parameters are set as *n* = 1.0, *n* = 1.46, and *n* = 1.6. Due to the fact that the reflection coefficients *R*_1_ and *R*_2_ are stable, the fringe visibilities of the interference spectra are around 15 dB in various RIs. These results show that the proposed FFPI sensor can maintain a high signal intensity and a clear fringe visibility on a wide RI measurement range.

According to Equations (1) and (2), the normalized total reflective intensity *I_Rtotal_*(*λ*) reaches its minima when the phase of the cosine term *ϕ* becomes an odd number of π. As shown in Figure 2, the two adjacent interference minima *λ*_1_ and *λ*_2_ have a phase difference of 2*π*. If either the RI of measuring medium, *n*, or the length of the micro-cavity, *l*, is known, the other one can be calculated as follows [35,36]:

(4) $$l\cdot n=\frac{\lambda_{1}\lambda_{2}}{2(\lambda_{2}-\lambda_{1})}$$

**Figure 3:**
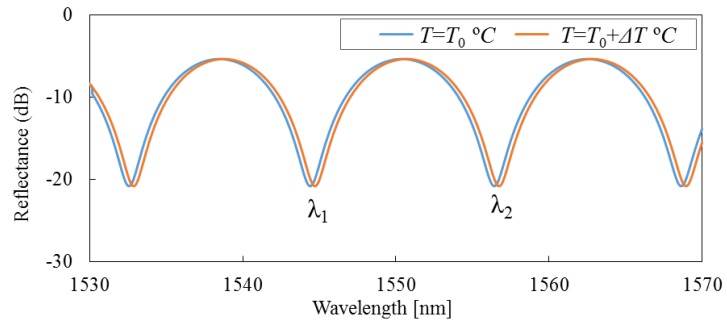
Comparisons of the interference spectra at a temperature difference *ΔT* = 500 °C, where all other parameters remain unchanged, that is *R*_1_ = 0.1, *R*_2_ = 1.0, *α* = 0.5, *β* = 0.5, *l* = 100∙[1 + *C*·(*T* − *T*_0_)] µm, and *C* is the linear thermal expansion coefficient of silica fiber, set as 4×10^−7^ /°*C*.

Equation (4) is used to calculate the RI of the measuring medium, assuming that *l* is kept constant during the measurement. In practice, because the linear thermal expansion coefficient of pure silica is about 4.0 × 10^−7^ /°C at room temperature [25,26], the length of the micro-cavity increases as the temperature rises. For instance, Figure 3 shows the effect of a temperature change on the position of interference spectra at a temperature difference of 500 °C, where *n* = 1.0 and *l* = 100 µm at the initial temperature. It can be seen that the interference spectra drifts to longer wavelengths as the temperature increases; therefore, *λ*_1_ and *λ*_2_ are variables with temperature. The error of the calculated RI is about 4.0 × 10^−7^ RIU/°C. Therefore, Equation (4) cannot be used directly to calculate the RI of the measuring medium at various temperatures.

In order to solve this problem, the length change of the micro-cavity has to be compensated for the measuring results of the RI. Thus, a new formula is proposed in this work based on the linear thermal expansion coefficient of optical fiber, and is given by:

(5) $$n=\frac{\lambda_{1}\cdot \lambda_{2}}{2l\cdot \left[1+C\cdot (T-T_{0})\right]\cdot (\lambda_{2}-\lambda_{1})}$$

where *C* is the linear thermal expansion coefficient of silica, *T* is the measurement temperature, *T_0_* is the initial temperature, and *l* is the initial length of the micro-cavity. Research results of our team and others have confirmed that, at various temperatures, the length change of the micro-cavity can be computed based on *l* ∙ [1 + *C* ∙ (*T* − *T*_0_)]; thus, the effects resulting from the temperature change can be eliminated [25,26,35,36]. Therefore, the RI can be measured accurately using Equation (5) at different ambient temperatures.

In some specific research areas, the RI variation of the measuring mediums is very small. When phase shift of the interference spectrum is less than 2π, since the change rate of RI equals to the change rate of the wavelength, the relative change of RI can be calculated by Equation (6) as given below [4,6]:

(6) $$\Delta n=\frac{\Delta \lambda_{i}}{\lambda_{i}}n_{0}$$

where *n*_0_ is the initial RI of the measuring mediums in the micro-cavity, *λ_i_* is the initial wavelength corresponding to any one of the interference minima, and *Δλ_i_* is the drift of *λ_i_* when *n*_0_ changes to *n*_0_ + *Δn*. Usually, the optical telecommunication wavelength region (around 1550 nm) is used to monitor the phase shift of the interference spectrum. Therefore, the solution of the relative RI value by Equation (6) has higher sensitivity and better resolution. It is noted that, for both evaluation methods, the actual RI sensitivity and resolution are highly dependent on the overall performances of the optical system, such as the wavelength resolution, stability and accuracy.

## 3. Experimental Verifications

### 3.1. Production and Evaluation of Gold Thin Films 

In this study, the gold thin film of various thicknesses is used as an STM or a TRM. A gold sputtering process is applied to fabricate vertical mirrors for optical applications. The film thickness is controlled by sputtering time in our work [31,32]. The gold film-forming speed of ANELVA SPP-430H (partial transformation) (Canon, Kawasaki, Japan) is 8.3 nm/min. To evaluate the correlation between optical parameters and film thickness, three gold thin films with 5 nm, 10 nm and 100 nm thickness are prepared in the Corning® SMF-28e optical fibers (New York, NY, USA), and evaluated through experiments.

**Figure 4:**
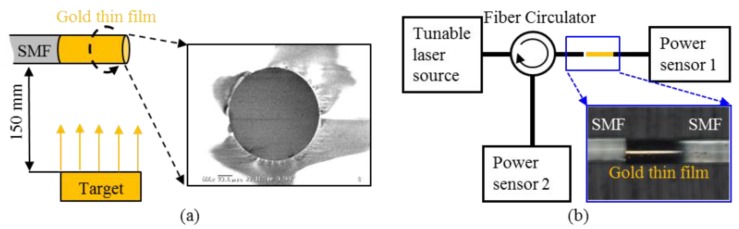
Production and evaluation of the gold thin films (**a**) scheme of sputtering system and SEM image of the fabricated gold thin film using sputtering process; (**b**) measurement system for the transmittance and reflectance of the gold thin film.

The fabrication process of the gold thin film is as follows. (1) Remove the coating of the SMF. (2) Cut the SMF to obtain a flat surface using the fiber cutter. (3) Wash the SMF using ethanol followed by air blowing. (4) Sputter gold thin film. The scheme of sputtering system and the scanning electron microscope (SEM) image of the gold thin film is shown in Figure 4a. Then, the transmission coefficients and the reflection coefficients of the gold thin films with different thicknesses are evaluated. The experimental system setup is shown in Figure 4b. A tunable laser (Agilent 81600B #201, Santa Clara, CA, USA) is used that is continuously swept from 1530 nm to 1570 nm at intervals of 10 pm wavelength. The continuous wave (CW) laser light is injected into an SMF through a fiber circulator, which is connected to a coating SMF by gold. The transmitted light is received by power sensor 1. In addition, the reflected light once again passed through the input SMF and is received by power sensor 2.

**Figure 5:**
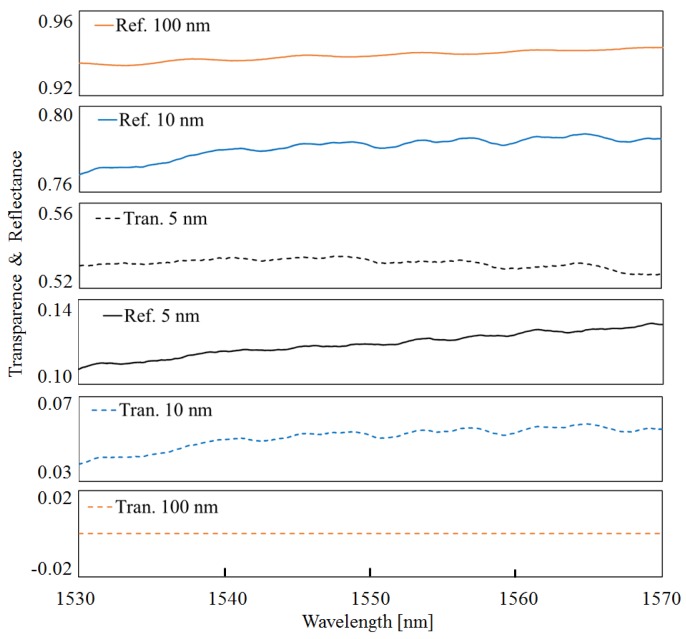
Spectra of the transmittance and reflectance of the gold thin films with various thicknesses.

As shown in Figure 5, the reflectance of the 100 nm thick gold thin film is about 0.94, and the transmittance is zero in the wavelength range of 1530 nm to 1570 nm. Thus, a gold thin film of definite thickness can be used as a TRM. It is also shown that the reflectance decreases and the transmittance increases when the gold thin film is getting thinner. The reflectance of the 50 nm thick gold film is about 0.1, and the transmittance is about 0.5, respectively. Therefore, the gold film of suitable thickness can be used as an STM. These optical properties are used at the proposed FFPI sensor.

### 3.2. Fabrication of Sensor Head

**Figure 6:**
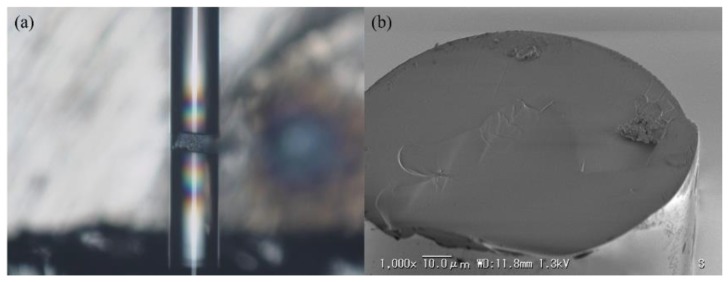
Images of the fabricated micro-cavity. (**a**) a SMF with a micro-cavity. (**b**) the SEM image of the inner surface of the micro-cavity. The machined surface by FIB milling is shown at the upper half of the fiber’s fracture surface.

Another key structure of the sensor head is the opened micro-cavity. It is fabricated using focused ion beam (FIB) milling technology in this work. In this study, the FFPI sensor head is made of an SMF-28e. The diameter of the optical fiber is 125 µm and the diameter of the core is 8.2 µm. To test the roughness of the machined surface, a micro-cavity is fabricated by Ga ions using an FEI FIB 500 system (FEI Company, Hillsboro, OR, USA). The Ga liquid ion gun is set to 25 kV, producing a high beam current of 6.4 nA. An SMF with a 40 µm’s long micro-cavity is shown in Figure 6a. It is broken to examine the machined surface. The SEM image shows that a smooth machined surface can be obtained using FIB as indicated in Figure 6b.

Then, a micro-cavity with 100 × 80 µm^2^ is opened at the lateral face of optical fiber by FIB milling, and the gold thin films are fabricated by vacuum sputtering. The fabrication process is shown as follows. (1) Remove the coating of the SMF. (2) Cut the SMF to obtain a flat surface using the fiber cutter. (3) Wash the SMF using ethanol followed by air blowing. (4) Machine a micro-cavity by FIB milling. The time of milling is set to be 70 h. (5) Sputter 95 nm thick gold thin film. The time of sputtering is set to be 11.5 min. (6) The gold film on the left is removed by FIB milling. The time of milling is set to be 10 min. (7) Sputter 5 nm thick gold thin film. The time of sputtering is set to be 36 s.

**Figure 7:**
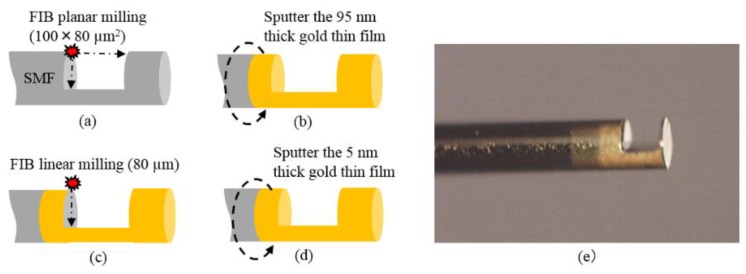
The schematic diagram of key procedures. (**a**) The micro-cavity is machined by FIB. (**b**) A 95 nm thick gold thin film is sputtered. (**c**) The gold film on the left of the micro-cavity is removed by FIB milling. (**d**) A 5 nm thick gold thin film is sputtered. (**e**) Image of the fabricated sensor head.

The main process and the micrograph of the fabricated sensor head are shown in Figure 7. The length between TRM and the endface of the fiber is only 10 µm. This type of structure can provide suitable mechanical strength and measuring stability by the research results of Pevec *et al.* [29]. With such a short structure of TRM, sensor head breakage is unlikely to happen by external forces from measuring medium flow.

### 3.3. Experimental Results

**Figure 8:**
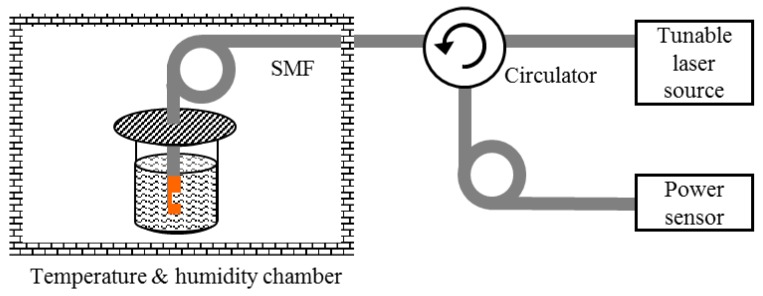
Experimental setup.

The experimental setup is shown in Figure 8, consisting of a fiber circulator, a power sensor, and a tunable laser that is continuously swept from 1530 to 1570 nm at intervals of 1.0 pm wavelength. The sensor head is placed into a high-performance temperature and humidity-controlled chamber (temperature uniformity: ±0.1 °C) with a temperature range from −20 to 80 °C.

**Figure 9:**
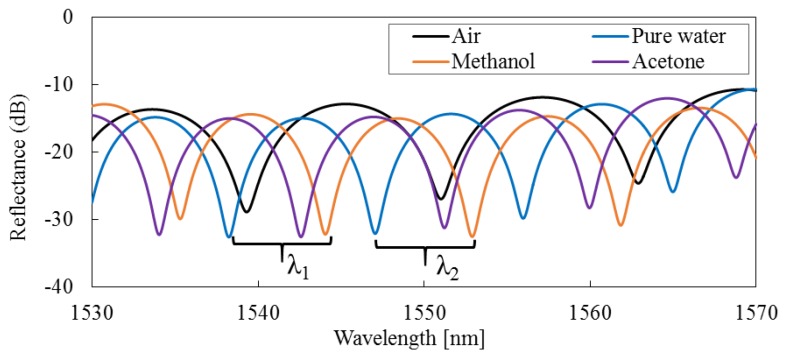
Interference spectra of the fabricated FFPI sensor in air, pure water, methanol, and acetone.

Firstly, the actual length of the machined micro-cavity is evaluated. In parameter settings of the FIB milling fabrication process, the length of the micro-cavity is set to be *l* = 100 µm. However, the errors in the machining dimension should be recognized. Since it is difficult to directly measure the length of the micrometer level, the actual length of the micro-cavity is obtained through rigorous calculations. The response of the sensor is investigated in air with *n* = 1.0003, while the temperature is kept at 20 °C. In addition, the environmental test chambers are held at 20% RH. The interference spectrum is shown in Figure 9, with *λ*_1_ = 1539.327 nm and *λ*_2_ = 1551.033 nm. Therefore, the length of the micro-cavity can be computed effectively by Equation (4); here, *l* = 101.949 µm length. The deviation of the actual length from its set processing length is about 2%. In the steps that follow, the capability of the fabricated FFPI sensor is evaluated using this actual length of the micro-cavity.

Next, to evaluate the capability of the fabricated FFPI sensor, the RIs of pure water, methanol, and acetone are measured. The interference spectra of the sensor head immersed in various liquids are also shown in Figure 9. In addition, the fringe visibilities of optical interference are more than 15 dB and stable signal intensities and fringe visibilities are maintained. Two adjacent interference minima and demonstrated RIs are listed in Table 1. Using Equation (5), the refractive indices of the liquids are calculated to be: *n_Pure water_* = 1.3245, *n_methanol_* = 1.3295, and *n_acetone_* = 1.3585, which are the commonly accepted values.

As stated in Section 2, the actual RI sensitivity and resolution are highly dependent on the overall performances of the optical system. In the above experiments, a tunable laser source with continuously sweeping intervals at 1.0 pm wavelength is used. The RI resolution of the fabricated FFPI sensor with 1.0 × 10^−4^ RIU is proved by Equation (5) in our work. Moreover, the relative change of the RI can also be calculated by Equation (6) when the RI variation of the measuring mediums is small. As shown in Table 1, interference minima *λ*_1_ and *λ*_2_ change for different RIs of methanol and pure water. Thus, *Δn* is obtained as the difference between the RIs of methanol and pure water and is equal to 0.005 RIU. *Δλ*_i_ is obtained as the difference between interference minima of methanol and pure water and is equal to 5.811 nm and 5.844 nm, *I* = 1, 2. The sensor sensitivity is then calculated as the ratio between *Δλ*_i_ and *Δn* and is equal to 1162.2 nm/RIU and 1168.8 nm/RIU, respectively. Moreover, RI resolution of the fabricated FFPI sensor is equal to the ratio of the sensor sensitivity to the wavelength resolution of the optical system; thus, RI resolution shows a smaller value than 1.0 × 10^−6^ RIU with a wavelength resolution of 1 pm.

**Figure 10:**
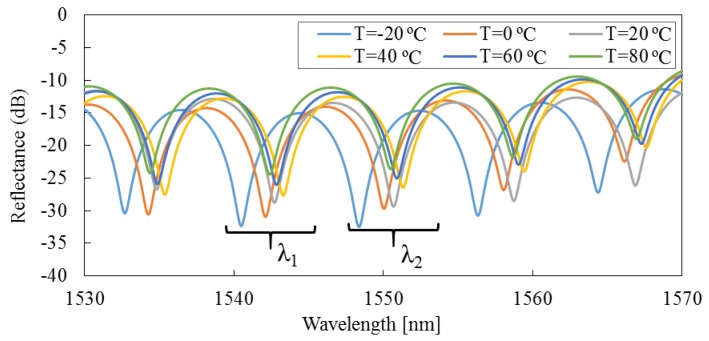
Interference spectra of the fabricated FFPI sensor in RIMM with temperature increase from −20 to 80 °C.

**Figure 11:**
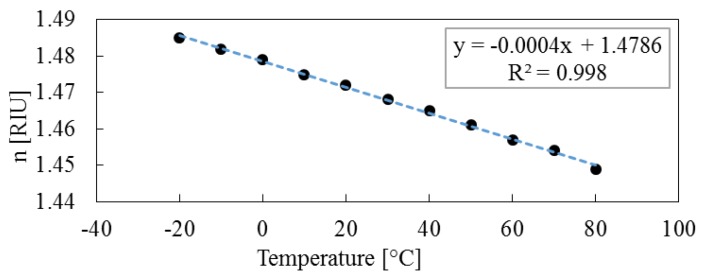
The computed RIs of RIMM in the temperature range from −20 and 80 °C.

Finally, the fabricated FFPI sensor is evaluated using a type of refractive index matching material (RIMM). It is known that the RI of RIMM is a function of temperature, and it changes linearly from 1.49 to 1.45 RIU with the temperature increasing from −30 to 80 °C by Data Sheet of Shin-Etsu Chemical® (Tokyo, Japan) [37]. In this study, the environmental test chambers are held at 20% RH, with temperature increment steps up of 20 °C from −20 °C to 80 °C. The interference spectra are shown in Figure 10. In the end, the RI of RIMM at various temperatures are computed by Equation (5), the temperature response (temperature increment step up of 10 °C) is shown in Figure 11. The measured results of the RIMM’s RI using the fabricated sensor agree well with the data given by the manufacturer.

## 4. Discussion

The interference spectra of the sensor head immersed in various measuring medium are shown in Figure 9 and Figure 10. The fringe visibilities of optical interference are higher than 15 dB, and the max variation of the fringe contrast is over 20 dB. These research results show that the proposed structure of the FFPI sensor can maintain a high signal intensity and a clear fringe visibility on a very wide measurement range of the RI. However, the interference spectra have also shown transmission losses of about 12 dB. The main reason is that the proposed FFPI sensor has an opened micro-cavity with about 100 µm in the optical fiber. Thus, the transmission loss is inevitable due to the light beam divergence in the micro-cavity. Although the beam divergence loss can be improved by reducing the length of the micro-cavity, the evaluation of interference spectrum needs to be done with wider wavelength range rather than the existing 1530 to 1570 nm. By doing so, the optical system based on broadband tunable laser source or optical spectrum analyzer (OSA) is very expensive. Contrastively, the wavelength tuning range of about 50 nm can be achieved by the low cost sample grating distributed Bragg reflector (SG-DBR) laser in practical applications. Thus, the length of micro-cavity is set to 100 µm in this study.

In Figure 9 and Figure 10, the non-flat baselines of interference spectra are also observed. The output power increases toward longer wavelengths, and the maximum peak difference is about 3 dB within the 1530 to 1570 nm wavelength range. This phenomenon mainly arises from the misalignment of the optical axis, which is a disadvantage of FIB milling technology. In this study, to obtain a high milling rate, the Ga liquid ion gun is set to 25 kV, producing a high beam current of 6.4 nA. In addition, the beam semiangle is 10 mrad [38,39], so the STM and TRM based on the inner surfaces of the micro-cavity are not vertical mirrors for the optical axis and have a slight angle deviation as 10 mrad. Accordingly, the output power increases toward longer wavelengths by coupling changes between the LP_01_ mode in the optical fiber and the light beam in the micro-cavity. Although it has little effect on the experimental results, optimizing the FIB milling process will be carried out in order to obtain better measurement performance in the next phase.

## 5. Conclusions

In this study, we have proposed and demonstrated a new type of FFPI sensor for the RI measurement. It has a special structure constituted by adding an STM and a TRM on a micro-cavity. The experimental results have shown that the proposed FFPI sensor can maintain a high signal intensity and a clear fringe visibility on a wide RI measurement range. Using two different evaluation methods, RI resolution better than 1.0 × 10^−4^ RIU on a wider RI measurement range for the fabricated sensor is proved through a calculation of the absolute RI. In addition, the sensor shows RI sensitivity higher than 1160 nm/RIU, and the RI resolution is better than 1.0 × 10^−6^ RIU when the RI change is small and the phase shift is less than 2π. Furthermore, the proposed sensor shows many desirable characteristics, such as good reliability, small size, easy operation, wide RI measurement range, and free flowing of the measuring medium due to the open micro-cavity. Thus, these types of FFPI sensors are able to be applied to many fields, including biochemical sensing and environmental monitoring. On the other side, although femtosecond laser micromachining of fiber-optic sensors has been reported in recent years as another good candidate for mass production and easy fabrication, various practical issues of the process such as reliability, stability, and lifetime effects are still under investigation. The main purpose of our work is to propose a new type of structure of the FFPI sensor and verify its feasibility. Therefore, FIB milling technology is still chosen in this study, although the machining time and cost of it are relatively high. Nevertheless, femtosecond laser micromachining techniques should still be further developed for a practical device to ensure mass production and a low cost in further studies.

## Figures and Tables

**Table 1:** Values of two adjacent interference minima and values of the RIs (at 20 °C)

Medium	Air	Pure Water	Methanol	Acetone
*λ*_1_ [nm]	1539.327	1538.243	1544.054	1542.585
*λ*_2_ [nm]	1551.033	1547.055	1552.899	1551.224
*l* [µm]	101.949	-	-	-
*n* [RIU]	-	1.3245	1.3295	1.3585
